# The Effect of Recreational Swimming on the Health of Students with Poor Somatic Health in Physical Education Classes at University

**DOI:** 10.3390/jfmk4030059

**Published:** 2019-08-20

**Authors:** Olena Dorofieieva, Kseniya Yarymbash, Iryna Skrypchenko, Ratko Pavlović, Georgian Badicu

**Affiliations:** 1 Department of Physical Rehabilitation and Sport Medicine, Bogomolets National Medical University, 01601 Kyiv, Ukraine; 2 Water Sports Department, Prydniprovsk State Academy of Physical Culture and Sport, 49094 Dnipro, Ukraine; 3 Faculty of Physical Education and Sport, University of East Sarajevo, Pale 71420, Republic of Srpska, Bosnia and Herzegovina; 4 Department of Physical Education and Special Motility, Faculty of Physical Education and Mountain Sports, Transilvania University of Braşov, 500068 Braşov, Romania

**Keywords:** students, physical rehabilitation, healthy swimming

## Abstract

Background: The physical education of students who have a deviation in their state of health requires a joint effort from teachers and doctors. Aim: The aim of the study was to substantiate the necessity of swimming classes as an effective means of physical rehabilitation in students with health disorders within the physical education curriculum classes. Methods: Students with low-level somatic health (54 students) were grouped into the Basic Group (BG, 27 students) and the Control Group (CG, 27 students). The Basic Group students were offered special swimming classes aimed at their physical rehabilitation. At the beginning of the study and after 24 training classes the authors assessed the somatic health, physical and mental endurance, and adaptation abilities of the autonomic nervous system. Results: Implementation of the method into the curriculum of the BG students resulted in a significant improvement (by 48.1%) of their somatic health. A reliable re-distribution of the students with “poor” and “lower than average” somatic health to the “average” and “higher than average” health group was noted (*p* < 0.05). The students’ physical characteristics improved by 36.4%. Conclusion: The conducted research proved the necessity of using sectional swimming activities as a means of physical rehabilitation of students with low health.

## 1. Introduction

University studies are associated within creased stress on students. Due to the extensive volume of knowledge which must be mastered, the students become less mobile, with hypodynamic and increasing static tension. Insufficient movement activity and hypokinesia, combined with insufficient physical loading, lead to the development to numerous diseases [1,2,3,4,5].

According to the Ministry of Health of the Ukraine, over last 5 years the number of healthy students has decreased by five times, and the total number of healthy freshmen does not exceed 10%. The level of total morbidity of the students under 21 years in 2015 exceeded 60%, of which the morbidity of students aged 15–17 years made up 48% [6,7,8,9].

Recently we have observed an expressed increase in the incidence of neuro-psychic disorders, and the psychic adaptation of the students has been aggravated, often leading to alcohol, tobacco, and drug abuse. The proportion of students diagnosed with several diseases is increasing: students aged 17–18 years old are diagnosed with 2–3 disorders on average, while those aged 19–22 years have four disorders, and graduates may be diagnosed with six or more functional disorders and chronic diseases [10,11,12,13].

However, it is worth mentioning that the student health deterioration is quite often caused by irrational and incorrect physical education classes, as these classes should presumably be aimed at dealing with health problems of the students [3]. In human health, 50% depends on life style, 20% on environmental conditions, 20% on heredity, and 10% on healthcare, i.e., causes which do not depend on the individual [14,15,16,17,18].

According to the statistics, it is possible to state that health education in university does not “appeal” to school authorities and teachers. Physical education of students with health problems, poor physical parameters, and poor physical fitness requires a combination of teacher and physician efforts [19,20,21]. Active motion activity is unavailable for students with poor health in the majority of cases, so a problem in satisfying this need may arise. Here, hypodynamics lead to even greater functional and morphological metamorphoses in the body [3,22,23].

Correct management of physical education classes conducted with the students will provide for strengthening their health and increasing the resistance to negative environmental factors, which, in turn, is the most effective method of non-specific prevention and the most important somatic health stimulator.

According to experts, one of these types of classes is recreational swimming, the main benefits of which are: maintaining the achieved level of health, maintaining a minimum level of physical activity to reduce the risk of cardiovascular diseases, increasing the functional reserves of the body, and learning the basics of swimming techniques [24,25].

Works by many authors have shown the positive impact of swimming on the physical and functional state of healthy students at universities [26,27,28] and in other population groups [29,30,31,32,33,34].

In the opinion of many authors, recreational swimming positively influences the indicators of students’ objective health that manifest in normalization of their cardio vascular systems and also positively influence the subjective health of trainees: claims of vegetative disorders and psycho-emotional de-adaptation reduce, and the self-estimation of one’s own health becomes more adequate [35,36,37,38,39,40].

Studies of different health related problems and life quality with the help of swimming have been presented in works of Ukrainian and foreign experts, where they showed directions of effective solution of mentioned problems and rendered practical recommendations for students with different levels of health [41,42,43,44,45,46,47].

The aim of this study was to substantiate the necessity of swimming classes as an effective means of physical rehabilitation in students with health disorders, within the physical education curriculum classes.

## 2. Materials and Methods 

### 2.1. Subjects

The study was held with 72 students from Bogomolets National Medical University, and 56 students from Dnipropetrovsk Medical Academy. In total, 128 students of the “special” (Ukrainian term, means those with poor health) medical group participated in the study. The average ages of the students ranged from 17 to 20 years (18.48 + 1.86). The students with “poor” and “poorer than average” health level (54 students) were divided between the Basic Group (BG) with 27 students and the Control Group (CG) with 27 students. The Basic Group criteria were as follows: recommendation of the physician, present health disorders (chronic diseases in remission, except for urinary tract pathologies); student’s wish to improve health and go swimming; ability to swim for 50 m with any possible swimming method; and an informed consent form signed by the student. The study was held in accordance with the scientific research theme of the Bogomolets National Medical University’s Physical Rehabilitation and Sports Medicine Department: “Life quality and physical health of the youth related to everyday movement activity”. In addition, the informed consent of the respondents was requested through a document in which the nature of the study was detailed.

### 2.2. Experimental Construct

The students of the Basic and Control Groups attended classes in physical education with the aim of improving health twice a week for 12 weeks.

The Basic Group students were offered swimming section classes. Throughout the studies (in early and late study period) the following criteria were detected: the infectious index, somatic health level, and physical health level, as well as the autonomic nervous system functional properties.

### 2.3. Method of Swimming Classes

The Basic Group students were offered to join the swimming section classes. 

The curriculum included 24 training classes (two educational-training classes a week, according to the timetable), which were divided into the water- and non-water classes. Each class lasted for about 80 min and included 20 min of warm-up and 60 min of swimming. The non-water classes included managing the posture and flat-foot drills, aimed at improving physical health level, etc. To improve the respiratory and cardiovascular health the authors used respiratory exercises. To increase the muscle and joints, elasticity–flexibility and stretching exercises were used. The authors also offered exercises aimed at learning swimming techniques to be practiced in a non-water environment. Water classes fulfilled the same tasks. The curriculum water cases provided for increasing adaptation abilities of the students with “poor” and “lower than poor” somatic health levels to physical loading of various types, sparing the cardiovascular system, external respiration system and muscle energy supplying. The offered tasks included swimming for 25–400 m distance, with the heart rate rhythm control. The authors observed post-exertion recovery and general self-feeling of the students with “poor” health. The loading was differentiated due to the exercises intensity, swimming speed varying with different distances and the intra-exercise intervals, according to the somatic health and initial physical health and endurance of the students. 

### 2.4. Infectious Index

Defining the infectious diseases morbidity rate throughout the year.

### 2.5. Evaluation of the Health Students Somatic by the G.L. Apanasenko Method

This included measuring and interpreting the anthropometric (body weight, height, power dynamometry), physiological (lung respiratory capacity hearth rate, blood pressure), and functional data (Martine–Kulishevskyi test), which were used for calculating the morphological–functional indices. The somatic health was evaluated by the total amount of points, which corresponded to the calculated parameters and defined functional classes, ranging from the “poor” to “very good”. 

### 2.6. Evaluation of Physical Health and Endurance of the Students

The authors express-assessed physical health and endurance by 11 criteria: age according to the passport; standard body weight appropriate to the age, gender and height of the respondents; standard arterial pressure according to the gender, body weight, and height; resting pulse; flexibility (bending down and touching the established region below and above the zero point); speed (the “race test” measuring speed of grasping the falling ruler which is falling with a stronger hand); dynamic power (maximum height of jumps in height); speed endurance (maximum frequency of raising the straightened legs up to 90° angle, with the patient supine with hands behind the head, during 20 s); speed-power endurance (maximum frequency of bending arms, the patient in a prone position, during 30 s); general distance endurance (running during 10 min (measuring the distance) and running for 2000 m (measuring the time)); and pulse recovery after 20 squats during 30 s. According to the obtained data we assessed the level of physical readiness to exercises: excellent, better than average or good, average, poorer than average, and poor.

### 2.7. Variation Pulsometry—Evaluation of the Regulation Mechanisms Strain

The electrocardiogram (ECG) was recorded continuously, without interruptions, on lead II for 2–3 min. After measuring the R-R intervals (in mm) (not less than 100 intervals), the dynamic row was defined, which was statistically processed to calculate the following parameters: Mo (Mode), AMo (Absolute Mode), ∆X, AMo/∆x.

The obtained data provide for calculation of the strain index (SI) which characterizes the degree of the blood flow regulation mechanisms functional strain:

SI = AMo/2 Mo·∆X

According to the received SI parameters, the following heart rhythm regulation functional states have been established:

- Standard. The registered SI parameter ranges within 50 to 200.
- Prevalence of the sympathetic nervous system activity SI ≥ 200.
- Prevalence of the parasympathetic nervous system activity SI ≤ 50.

Continuous ECG recording was conducted using the ECG complex “Cardiocom, Cardiolab” by Cardiolab SE (National Aerospace University named after N.E. Zhukovskiy “KhAI” Technical Condition33.1. Ukraine, 2014).

### 2.8. Statistical Analysis

The obtained results were statistically processed using the Statistica 10.0 software (StatSoft Russia is the official representative of the copyright holder of the Statistica series software products from TIBCO, USA).

The characteristics are represented as the average mean ± standard deviation (SD). To check the standard distribution of variables used an online calculator in Statistica 10.0. To determine the consistency of experimental data with the hypothesis of the normal distribution of random variable X, the Pearson’s test was applied. The actual observed value was Χ^2^_obs_ = 1.373. The number of intervals was *q* = 5, and the normal law of distribution was determined by two parameters, which we estimated by sampling and then the number of degrees of freedom. Behind the table Χ^2^_cr_(0.05;2) = 5.99. Due to the fact that Χ^2^_obs_ < Χ^2^_cr_, then the hypothesis of a normal law of distribution of a random variable X agrees with the experimental data and is not rejected. Logarithm transformation was conducted in case of abnormal distribution. A two-sided repeated assessment was used to compare the data obtained during and in the late swimming section program introduction period. The reliability of the obtained changes was determined by Wilcoxon criterion, the reliability criterion was *p* < 0.05.

## 3. Results

Estimation of the somatic health of the first and second year students showed a prevalence of the “average” somatic health level in 45 students (35.1%). The level termed as “better than the average” was characteristic for 19 students (14.0%) and “excellent level”for 10 students (7.8%). Fifty-four students (42.2%) showed “poor” and “poorer than average” somatic health levels (17.9% and 24.3% of the students, respectively).

The infection index in the examined students corresponded to the average one (2.84). During the examination they did not have health complaints, but a third of the students (33.6%) showed a high infection index (more than three infectious diseases episodes a year). These were presumably students with somatic health defined as “poor” and “poorer than average” (*p* < 0.05).

The assessment of physical fitness (PF) defined prevalence of the “average” and “poorer than average” somatic health levels (28.9% and 39.9%, respectively), which may be attributed to low motion activity of the students in general. The students with “average” somatic health level were characterized with the average physical fitness level in 51 students (39.9%), “higher than the average” in 16 students (12.5%), and “excellent” in seven students (*p* < 0.05).

Due to the fact that physical and psychic load adaptation is realized presumably due to autonomic regulation, the vegetative tonus was studied by the variation pulsometry method. Among the examined students, 40.6% referred to a sympathotonic type, 35.2% to a normotonic type, and 24.2% to a vagotonic type, which provides evidence of disrupted adaptation mechanisms. The largest number of sympathotonic type students was determined among the students with “poor” and “poorer than average” somatic health levels.

To determine efficiency of the offered program and substantiate physical rehabilitation means the authors conducted a repeated testing of the somatic health and physical fitness with students of Control and Basic groups, which showed positive changes in the assessed parameters.

**Figure 1:**
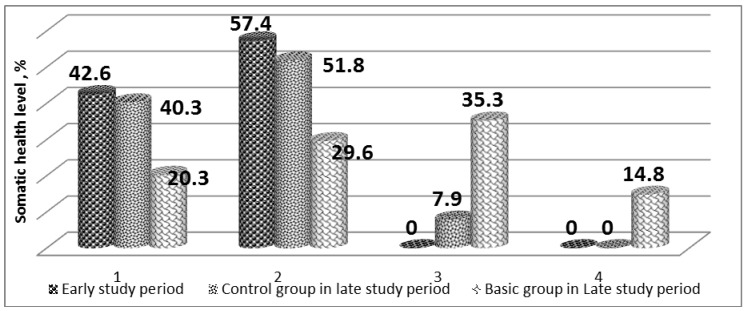
Dynamics of students somatic health level before and after the recreational swimming classes, % (*n* = 54, *n*1 = *n*2 = 27). Note: 1 “poor” level, 2—“poorer than average”; 3—“average”; 4—“better than average”.

The somatic health level reliably improved in the basic group (Figure 1). The number of students with “poor” and “poorer than average” somatic health level declined. Thus, “poor” somatic health level was revealed in five students (18.5%), while the “poorer than average” somatic health level was characteristic for 8 (29.6%). At early study stage these characteristics were shown in 42.6% and 57.4%, respectively. Thus, an increase in the somatic health level has made up 48.1%, which was reflected in re-distribution of the students by the somatic health groups. After the study 10 students (37.1%) showed average somatic health level, and four students (14.8%) showed the “better than average” somatic health level. It is worth mentioning that before the study, the Basic Group did not include students with “average” and “better than average” somatic health levels. In the Control Group, after recreational swimming classes, no reliable changes in somatic health were detected. The differences within the Basic and Control Group data are reliable (*p* < 0.05).

**Figure 2:**
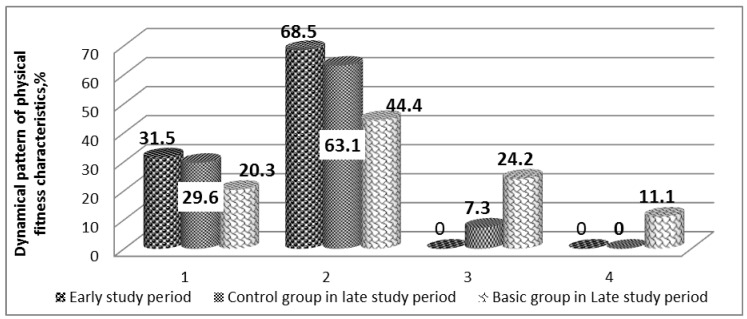
Dynamical pattern of physical fitness characteristics for the students with poor health before and after the recreational swimming classes, % (*n* = 54, *n*1 = *n*2 = 27). Note: 1—“poor level, 2—“poorer than average”; 3—“average”; 4—“better than average”.

The same reliably positive dynamics were established in assessment of the physical fitness levels (Figure 2). The number of the Basic Group students with “poor” physical fitness level decreased by 11.2%, and “poorer than average” physical fitness level by 22.1%. The physical fitness level increased in 10 students, from which seven at repeated testing showed an average physical fitness level, and three students reached the “better than average” level (*p* < 0.05). Altogether, no considerable changes were found in the Control Group.

The dynamical pattern of parameters which characterize the autonomic nervous system condition is the same. In the Basic Group, the number of sympathotonic group students decreased from 74.0% to 37.0% (*p* < 0.05), though the vagotonic and normotonic groups significantly increased. The Control Group did not show such a dynamic pattern.

## 4. Discussion

The study compares physical fitness data and adaptation abilities of autonomic nervous system as well as infectious index of students with “poor” and “poorer than average” somatic health levels.

As far as the authors are concerned, this is the first study which, based on the comparative analysis, provided for substantiation of the swimming section classes complex effect on the students youth health. 

The previous studies used individual data: dynamic pattern of the “special” medical group students’ somatic health changes [1,6,23] and their physical fitness [3,14,19,22,24]. These studies were related to traditional rehabilitation means introduced at section classes in special medical groups [14,24,25], and the details of these are absent. The studies of Hrubliak et al. [10] only state existing health disorders, and the works of Gladoshchuk and Sidorenko et al. reveal the effect of such physical education classes in students special medical groups on functional changes of basic functional systems [2,26]. 

Gryban offered general characteristic of students’ health [27,28], Redko et al. studied pedagogical aspects of health preservation and health-preserving technologies [29]. Hasimova [24], Sidorenko et al. [26], Drogomeretsky et al. [43], Mozgovoy and Donchenko [30] revealed the effect of individual physical rehabilitation methods applied within the university curriculum on the development of physical fitness, physical workability, and physical characteristics of the special medical group students.

An important factor in this study is that the offered method, being conducted in two stages (water and non-water stage) provides for complex simultaneous improvement of the functional characteristics, improving the cardio-vascular and respiratory system function, muscle-joint elasticity, adaptation properties of the body, and energy supply of muscle action, and, due to the swimming motion peculiarities (cyclic motions), increases the number of the students with normotonic autonomic nervous system regulation.

Previous studies [7,11,31,32,33] reported a quite small positive dynamic health pattern or its absence (neutral effect) in the special medical groups health condition as well as their physical fitness level. The conducted study provided for establishing reliable changes in all studied parameters.

Another distinct feature of the offered swimming classes method is a possibility of preventive rehabilitation. 

Despite the obtained results, the authors have revealed some limitations for the method, represented, firstly, with students’ poor ability of swimming for 50–100 m using any swimming style, and, secondly, with present urinary diseases in students.

## 5. Conclusions

The conducted study substantiated the necessity of swimming section classes held within physical education classes as a method of rehabilitation for students with “poor” and “poorer than average” somatic health levels, as confirmed with the received data.
